# Synthesis of AG@AgCl Core–Shell Structure Nanowires and Its Photocatalytic Oxidation of Arsenic (III) Under Visible Light

**DOI:** 10.1186/s11671-017-2017-9

**Published:** 2017-04-04

**Authors:** Yanyan Qin, Yanping Cui, Zhen Tian, Yangling Wu, Yilian Li

**Affiliations:** School of Environmental Studies, China University of Geosciences (Wuhan), Wuhan, 430074 China

**Keywords:** Arsenic (III), Photocatalytic oxidation, Ag@AgCl core–shell structure nanowires, Visible light

## Abstract

Ag@AgCl core–shell nanowires were synthesized by oxidation of Ag nanowires with moderate FeCl_3_, which exhibited excellent photocatalytic activity for As(III) oxidation under visible light. It was proved that the photocatalytic oxidation efficiency was significantly dependent on the mole ratio of Ag:AgCl. The oxidation rate of As(III) over Ag@AgCl core–shell nanowires first increased with the decrease of Ag^0^ percentage, up until the optimized synthesis mole ratio of Ag nanowires:FeCl_3_ was 2.32:2.20, with 0.023 mg L^−1^ min^−1^ As(III) oxidation rate; subsequently, the oxidation rate dropped with the further decrease of Ag^0^ percentage. Effects of the pH, ionic strength, and concentration of humic acid on Ag@AgCl photocatalytic ability were also studied. Trapping experiments using radical scavengers confirmed that h^+^ and ·O_2_^−^ acted as the main active species during the visible-light-driven photocatalytic process for As(III) oxidation. The recycling experiments validated that Ag@AgCl core–shell nanowires were a kind of efficient and stable photocatalyst for As(III) oxidation under visible-light irradiation.

## Background

As(III) is prevalent in anoxic groundwater, more harmful to human health, more mobile, and less efficiently removed than As(V); thus, pre-treatment by transforming As(III) to As(V) using different types of oxidation technologies is highly desirable for enhancing the immobilization of arsenic and is a prerequisite step for the use of sequent arsenic removal technologies such as coagulation, sorption, and membrane filtration. In recent years, photocatalytic oxidation technology has been considered as a potential and environmentally acceptable technique for As(III) oxidation [1]. Up to now, the photocatalyst TiO_2_ has been reported to be widely used for the oxidation from As(III) to As(V) in many literatures [2]. Rapid oxidation from As(III) to As(V) can be realized in TiO_2_ suspensions under UV irradiation [3]. The photocatalysis mechanism of TiO_2_ involves the generation of valence band holes and conduction band electrons under UV illumination and the subsequent generation of hydroxyl (HO·) and superoxide radical (·O_2_^−^) [4]. However, as we know, TiO_2_ crystalline phase can only be photoexcited by UV light (*λ* < 388 nm, a small fraction of solar spectrum) which only accounts for about 5% of the solar light energy owing to its large band gap (3.2 eV). This practically rules out the use of sunlight as a light source.

In order to expand the absorption band of TiO_2_-based photocatalysts, numerous attempts such as deposition with metal cations or nonmetal ions [5], decoration with another semiconductor [6, 7], and coating with organic matters [8] have been devoted to modify the photocatalyst TiO_2_. By far, TiO_2_-based nanoparticles functionalized with noble metal Pt nanoparticles [9] and sensitized with ruthenium dye [10, 11] have been used for As(III) oxidation, and all exhibited excellent photocatalytic oxidation performance for As(III) under visible light. Recently, visible-light driving photocatalysts were firstly used in As(III) oxidation by Hu et al. [12]; they found BiOI showed its great potential application value in photocatalytic oxidation of As(III). Later, Kim et al. firstly verified that tungsten trioxide (WO_3_) [13] was also active under visible light and could successfully oxidize As(III) to As(V).

Considering energy utilization and saving, the investigation of the development of visible-light-driven photocatalysts has currently been considered to be a hot topic. Recently, various Ag-based compounds such as silver/silver halides (Ag/AgX, X = Cl, Br, and I), Ag_3_PO_4_ [14], Ag_2_CO_3_, Ag_3_AsO_4_, Ag_x_Si_y_O_z_ [15], and Ag_x_(SiO_4_)_y_(NO_3_)_z_ [16] were demonstrated to be a new family of highly efficient visible-light photocatalytic materials. Among the Ag-based visible-light-energized photocatalysts, silver/silver chloride (Ag/AgCl) is one of the most attractive candidates that can meet the requirements of excellent visible-light absorption, high photocatalytic efficiency, and stability, owing to the surface plasmon resonance (SPR) characteristics of metallic silver nanoparticles (NPs) which can promote charge separation/transfer efficiently [17]. It is mainly used in water photolysis, water disinfection, pollutant degradation [18], carbon dioxide reduction, etc. Core–shell structured nanomaterials have aroused considerable attention in recent years because of their unique and tunable properties by changing either the constituting materials or the ratio of core to shell. Such adjustable structures with suitable shell materials enable these functional materials to meet application requirements in protection, modification, and functionalization of core particles [19].

One-dimensional (1-D) nanostructure-based catalysts such as wires, rods, and tubes [20] have been the focus of many recent studies due to their intrinsic large aspect ratio which favors a directional charge transport with a reduced grain boundary. The superior charge transport property of the catalytic species generally plays an important role for the enhancement of photocatalytic performances [21]. Among them, 1-D core–shell nanowires (NW) have recently become of particular interest because the function of the fabricated core–shell nanostructure can be further tuned or enhanced by coating the nanowires with a thin layer of another material and adjusting their core and shell with different materials [21]. As the consequence, considering the specific and excellent properties of 1-D core–shell nanowires, Ag nanowires were first used as chemical templates for the synthesis of 1-D Ag@AgCl core–shell nanowires by Bi and Ye [22] through an in situ oxidation reaction between Ag nanowires and FeCl_3_ solution, which exhibited excellent photocatalytic performance for the decomposition of methyl orange (MO) dye under visible-light irradiation. Ag@AgCl core–shell nanowires were also synthesized by etching Ag wires using ionic liquid [Bmim]FeCl_4_ IL and showed high visible-light photocatalytic ability [23]. Zhu et al. added Ag nanowires into H_2_O_2_ solution and then dropped hydrochloric acid into the above aqueous solution to synthesize Ag@AgCl core–shell nanowires [24]. AgCl nanowires decorated with Au nanoparticles by reducing Au precursors with Fe^2+^ ions could efficiently decompose methylene blue molecules under illumination of white light [25]. All these works illustrated that Ag@AgCl core–shell nanowires exhibited unique photocatalytic ability.

While As(III) photocatalytic oxidation performance using Ag@AgCl core–shell nanowires with different ratios of Ag nanowires:Ag^+^ was not researched, in this work, Ag@AgCl core–shell nanowires with different ratios of Ag: AgCl were synthesized to realize the rapid oxidation of As(III) under visible-light irradiation. Photocatalytic mechanism in this oxidation process for As(III) and recycling tests of Ag@AgCl core–shell nanowires were also studied.

## Methods

### Materials

Twenty milligrams per liter Ag nanowire dispersion was purchased from Xianfeng (China). Polyvinylpyrrolidone (PVP) was purchased from Sigma-Aldrich (USA), FeCl_3_ and EDTA were purchased from Aladdin (USA), high-performance liquid chromatography (HPLC) grade methanol was purchased from Fisher (USA) for HPLC analysis, and tetrabutylammonium hydroxide was purchased from PerkinElmer (America). One thousand milliliters of As(III) standard solution (1000 mg/L) was prepared by dissolving 1.3203 g of As_2_O_3_ in the minimum amount of 4.0 M NaOH solution and then adjusting pH to 3.0 with 1.0 M H_2_SO_4_ solution. All solutions and subsequent dilutions were prepared using deionized water from a scientific nanopure water purifier (Thermo fisher, America) with a resistivity of less than 0.055 μS/cm.

### Instruments

Morphological analysis was performed on a Hitachi SU8010 field-emission scanning electron microscope (FE-SEM) (Japan) with an acceleration voltage of 2 kV.

The X-ray diffraction (XRD) data was detected via a AXS D8-Focus X-ray diffractometer using Cu Kα radiation (Bruker, Germany). The operated conditions were controlled at 40 kV and 40 mA with a scan step width of 0.01°, and the scan range was 20°–90°.

X-ray photoelectron spectroscopy (XPS) measurements were carried out on a RBD upgraded PHI-5000C ESCA system (PerkinElmer, America) with Al X-ray source operating at 250 W. All the binding energies were referenced to the C 1 s peak at 284.8 eV of the surface adventitious carbon.

To detect concentration of As(III) and As(V), an ELAN DRC II inductively coupled plasma mass spectrometry (ICP-MS) (PerkinElmer, America) equipped with an atomizer and a spray chamber was used. The ICP-MS normal operating parameters were as follows: RF power 1100 W, lens voltage 7.25 V, nebulizer gas flow rate 0.98 L/min, auxiliary gas flow rate1.2 L/min, and plasma gas flow rate 15.00 L/min. Arsenic species were separated by Series 200 HPLC (PerkinElmer, America) with an automatic sample injector and directly introduced into ICP-MS. A C8 chromatographic column (PerkinElmer, America) was used with the mobile phase containing 1 mM tetrabutylammonium hydroxide, 0.05 mM dipotassium EDTA, and 0.05% methanol (pH 6.8).

### Preparation of Ag@AgCl Core–Shell Nanowires

Ag@AgCl core–shell nanowire dispersions with different mole ratios of Ag:AgCl were synthesized using an in situ oxidation method. In a typical procedure, 250 μL dispersion of 20 mg/L Ag nanowires was added to 10 mL deionized water in every 25-mL beaker. One hundred fifty microliters aqueous solution of 1 M PVP was added to the dispersion of Ag nanowires. The dispersion was vigorously stirred with a magnetic stirrer for 5 min before different volume of 20 mM FeCl_3_ solution was dropwise injected into the dispersion. The adding volume of 20 mM FeCl_3_ solution which was freshly prepared in order to avoid hydrolysis was 0.5, 1, 1.5, 2.0, 2.2, and 4.0 mL, respectively. The reaction solution was stirred for 1 h until the color of the solution became stable. The reaction of the Ag nanowires with different volume FeCl_3_ transformed them to Ag@AgCl core–shell nanowires with different mole ratios of Ag:AgCl. The resulting Ag@AgCl core–shell nanowires were centrifuged to remove excess FeCl_3_ and PVP after reaction. Every kind of Ag@AgCl core–shell nanowires was dispersed in 0.5 mL of deionized water after being washed by water and alcohol. The synthesis was carried out at room temperature and could be scaled up.

The quality of Ag@AgCl core–shell nanowires synthesized with 250 μL Ag nanowire dispersion and 2.2 mL FeCl_3_ solution was equal to 6 mg, and the mole ratio of Ag nanowires:FeCl_3_ used in the preparation of this kind of Ag@AgCl core–shell nanowires was 2.32:2.20. Subsequently, this kind of synthesized Ag@AgCl core–shell nanowire dispersion was prepared in a large scale according to the above process, then dried in the air at 50 °C for 8 h, and grinded to obtain the solid powder of Ag@AgCl core–shell nanowires with the mole ratio of Ag nanowires:FeCl_3_ = 2.32:2.20. The dry solid was used in the subsequent batch tests for influence factors, mechanism, and recycling.

### Photocatalytic Oxidation for As(III)

**Fig. 1:**
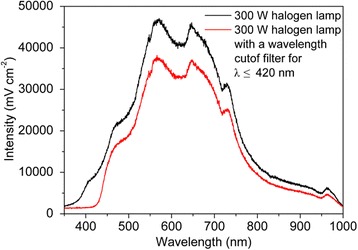
Spectral distribution of the 300 W halogen lamp with and without optical filter

Photocatalytic activities for As(III) oxidation were conducted in a 25-mL quartz beaker. The initial arsenite concentration of the solution with 0.02 mol/L Na_2_SO_4_ was fixed at 2.0 mg/L, and the pH was adjusted with H_2_SO_4_ or NaOH solution to 7.0. Prior to each As(III) oxidation, Ag@AgCl core–shell nanowires were added in the arsenite solution with a fixed volume of 20 mL. Prior to the irradiation, the suspension was kept in dark environment for 30 min to achieve adsorption/desorption equilibrium for arsenite. Then, the sample was exposed to 300 W halogen lamp equipped with a UV (less than 420 nm wavelength) cutoff filter, and the illumination intensity is shown in Fig. 1. In the whole photocatalytic process, air was aerated continuously through the suspension, which caused the reaction mixture to continuously stir. Water samples were withdrawn by a 0.4-mL pipette intermittently during photoreaction and filtered through a 0.22-μm PTFE filter. Duplicate or triplicate experiments were performed for each set.

To study the effect of the mole ratio of Ag:AgCl on photocatalytic oxidation for As(III), 0.5 mL suspension of Ag@AgCl core–shell nanowires with different mole ratios of Ag:AgCl for As(III) was respectively added in the 20 mL aqueous solution of 2.0 mg/L As(III).

In the subsequent batch experiments for influence factors, mechanism, and recycling, 6 mg solid of the Ag@AgCl core–shell nanowires synthesized with the mole ratio of Ag nanowires:FeCl_3_ = 2.32:2.20 was added in the 20 mL aqueous solution of 2.0 mg/L As(III) in every test.

## Results and Discussion

### Physiochemical Characterization of Ag@AgCl Core–Shell Nanowires

#### Morphology Study (SEM)

**Fig. 2:**
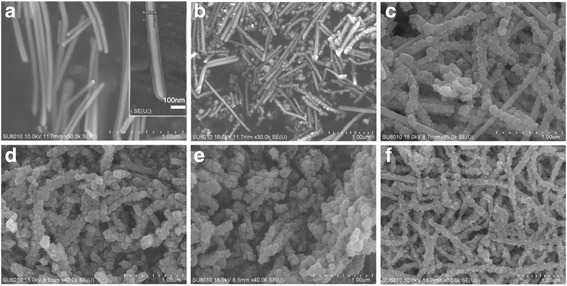
SEM images of **a** Ag nanowires and Ag@AgCl core–shell nanowires with different mole ratio of Ag:AgCl. Ag@AgCl core–shell nanowires were synthesized through the reaction between 250 μL Ag nanowire dispersion and **b** 0.5, **c** 1.0, **d** 1.5, **e** 2.0, and **f** 2.2 mL of 20 mM FeCl_3_ solution

Figure 2 presents a series of SEM images from samples containing 250 μL dispersion of Ag nanowires (20 mg/L) before and after they reacted with different volumes of 20 mM FeCl_3_ solution, clearly showing the morphological evolution involved in the conversion of Ag nanowires to Ag@AgCl core–shell nanowires. As shown in Fig. 2a, the Ag nanowires exhibited smooth surfaces and have diameters of 50–65 nm and lengths of 20–60 μm. After the addition of a small amount of FeCl_3_ (i.e., 0.5 mL), some defect sites with higher surface energies on the surface of Ag nanowires were decorated with cubic nanocrystals, which made the surface to become rough (Fig. 2b); this indicated that Ag atoms around the defects of the Ag nanowire were oxidized to Ag^+^ ions, and Ag^+^ ions immediately reacted with Cl^−^ ions to nucleate and condense into AgCl nanocrystals at these defect sites on the surface of Ag nanowires. As the consequence, cubic AgCl nanocrystals were formed on the surface of Ag nanowires, and the surface area of the composite became larger. With the increasing additional amount of FeCl_3_, more Ag atoms on the surface of Ag nanowires were converted into more AgCl nanocrystals (Fig. 2f), and the smooth surface of Ag nanowires gradually become the rough and thick shells of AgCl nanocrystals. It was observed that the surface area of Ag@AgCl core–shell nanowires became larger and larger with the mole ratio of AgCl. By theoretical calculation, 250 μL dispersion of 20 mg/L Ag nanowires totally changed to AgCl; 2.32 mL of 20 mM FeCl_3_ solution was needed. Figure 2f also proved that the whole surface of Ag nanowires were completely converted into AgCl nanocrystals with the addition of 2.2 mL FeCl_3_ solution, and the Ag nanowires thoroughly became the Ag@AgCl core–shell nanowires.

#### Phase and Compositional Study (XRD)

**Fig. 3:**
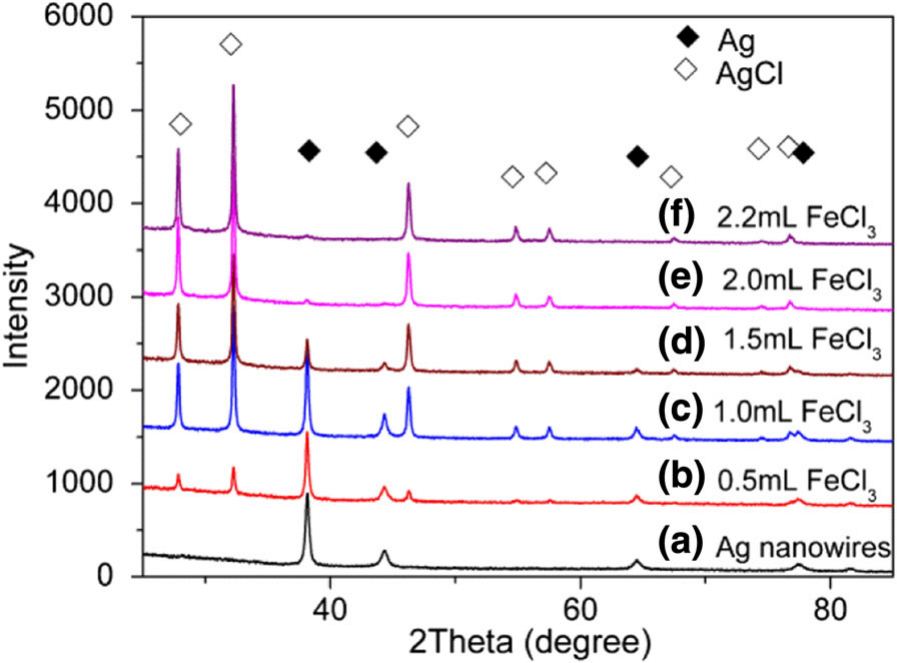
The XRD patterns of (*a*) Ag nanowires and Ag@AgCl core–shell nanowires with different mole ratio of Ag:AgCl. Ag@AgCl core–shell nanowires were synthesized through the reaction between 250 μL Ag nanowire dispersion and (*b*) 0.5, (*c*) 1.0, (*d*) 1.5, (*e*) 2.0, and (*f*) 2.2 mL of 20 mM FeCl_3_ solution

The corresponding XRD patterns of Ag nanowires were shown in Fig. 3a. The diffraction peaks at 2*θ* = 38.1°, 44.3°, 64.4°, and 77.5° marked with “black diamond” were assigned to the (1 1 1), (2 0 0), (2 2 0), and (3 1 1) planes of metallic Ag (JCPDS cards no. 04-0783). Figure 3b–f shows the XRD patterns of Ag@AgCl core–shell nanowires synthesized with different amount of FeCl_3_. Apart from the peaks which ascribed to metallic Ag crystals, the diffraction peaks at 2*θ* = 27.8°, 32.2°, 46.3°, 54.8°, 57.6°, 67.4°, 74.5°, and 76.6°, which were marked with “white diamond,” were assigned to the (1 1 1), (2 0 0), (2 2 0), (3 1 1), (2 2 2), (4 0 0), (3 3 1), and (4 2 0) planes of AgCl crystal (JCPDS cards no. 31-1238). With the increase of the additional volume of FeCl_3_ solution, the peak strength of metallic Ag gradually weakened after the oxidation reaction with FeCl_3_ and the peaks of AgCl crystals significantly increased. When the volume of FeCl_3_ solution was 2.2 mL, the SEM image (Fig. 2f) indicated that the surface of silver nanowires was almost completely covered by AgCl nanocrystals, and XRD diagrams showed that only a small amount of silver element still existed.

#### X-ray Photoelectron Spectroscopy (XPS)

**Fig. 4:**
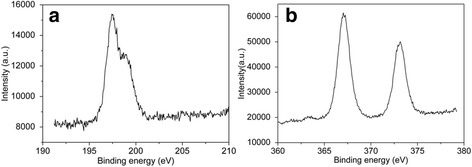
XPS spectra of **a** Cl 2p region and **b** Ag 3d region of Ag@AgCl core–shell nanowires synthesized with the mole ratio of Ag nanowires:FeCl_3_ = 2.32:2.20

XPS analysis was carried out to identify the chemical composition and binding states on the surface of Ag@AgCl core–shell nanowires synthesized with the mole ratio of Ag:FeCl_3_ = 2.32:2.20. It was shown from Fig. 4a that the XPS spectrum of Cl 2p displayed two bands at 197.3 and 199.1 eV, which could be ascribed to Cl (2p_3/2_) and Cl (2p_1/2_), respectively [26].

The XPS spectrum of Ag 3d in Fig. 4b showed that the 3d_5/2_ and 3d_3/2_ signals were located at 373.151 and 367.051 eV, respectively, and the splitting of the 3d doublet was about 6.0 eV. However, the two bands could not be divided into different peaks and were both attributed to the Ag^+^ of AgCl, which indicated that the peaks of metallic Ag centered at 368.73 and 374.91 eV were not contained in this XPS spectra, which meant that only AgCl crystals existed on the surface of Ag@AgCl core–shell nanowires.

### Photocatalytic Oxidation Experiments for As(III)

#### Photocatalytic Oxidation of Ag@AgCl Core–Shell Nanowires Synthesized with Different Mole Ratios of Ag Nanowires:FeCl_3_ for As(III)

**Fig. 5:**
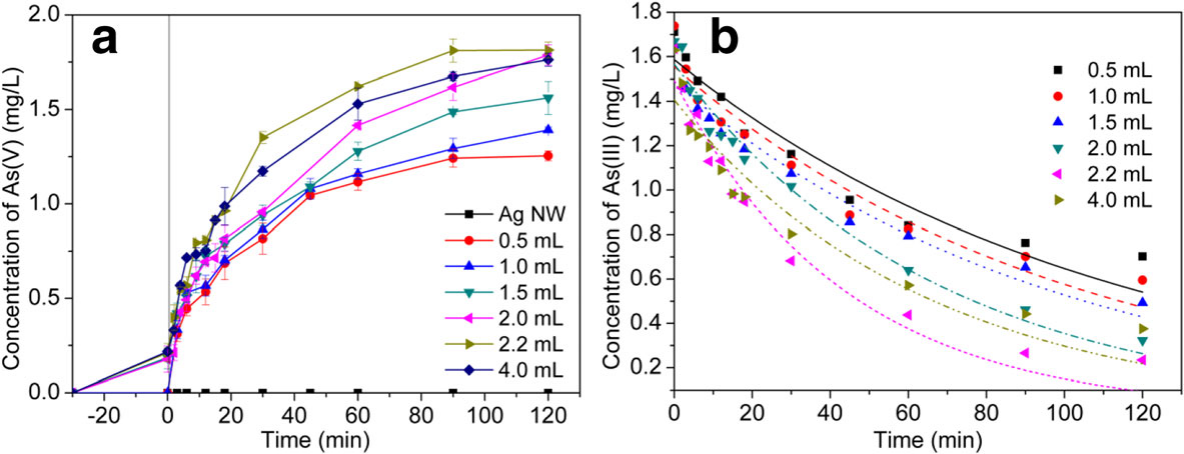
**a** Photocatalytic activities of Ag@AgCl core–shell nanowires for As(III) oxidation under visible-light irradiation. **b** Pseudo first order plot of As(III) photocatalytic oxidation by Ag@AgCl core–shell nanowires under visible-light irradiation

To evaluate the photocatalytic activity of Ag@AgCl core–shell nanowires synthesized with different ratios of Ag:AgCl for As(III) oxidation, the photocatalytic oxidation of As(III) was carried out in aqueous solution under visible-light irradiation (Fig. 5a). For comparison, As(III) oxidation was also performed in Ag nanowire suspensions under the same condition. Ag nanowires showed no photocatalytic activity with visible light in 120 min. However, when Ag nanowires were etched into Ag@AgCl nanowires by FeCl_3_, the transformation from As(III) to As(V) occurred (Fig. 5a), and it was clear that the photocatalytic oxidation rate of As(III) over Ag@AgCl core–shell nanowires increased with the rise of the mole ratio of AgCl first. The sample Ag@AgCl core–shell nanowires synthesized with the mole ratio of Ag nanowires:FeCl_3_ = 2.32:2.20 showed highest activity than other core–shell Ag@AgCl and pure Ag nanowires. Afterwards, the photocatalytic activity decreased with the further rise of the mole ratio of AgCl. This result was the same as the study of Ma et al., which also indicated that the photocatalytic activity of Ag@AgCl was significantly dependent on the mole ratio of Ag^0^:Ag^+^ on the surface of the catalyst, the optimum ratio being 0.035 [19]. The ratio of Ag and AgCl was a significant factor influencing the photocatalytic oxidation rate. When the amount of metallic silver was very scarce, the generation of the electron–hole pairs on the metallic Ag may be decreased due to the SPR effect on the metallic Ag, and the active species were also cut down, which could reduce the photocatalytic activity. But when the amount of Ag was too high, some of the Ag nanoparticles became the active sites promoting the recombination of the photoelectrons and the holes, which also reduced the photocatalytic activity [27].

Because of the strong photosensitive property of AgCl phase without Ag, under the sunlight, the photogenerated electron combines with an Ag^+^ ion to form an Ag^0^ atom; ultimately, a cluster of silver atoms is formed within a AgCl particle upon the repeated absorption of photons. So AgCl is instable under sunlight and seldom used as a photocatalyst. However, when a certain amount of Ag nanoparticles are deposited onto AgCl particles, electron–hole separation occurs smoothly in the presence of Ag nanoparticles, so metallic Ag plays an important role on the photoinduced stability of Ag/AgCl composite [28].

From the SEM images (Fig. 2), it could be observed that the surface area of Ag@AgCl core–shell nanowires became larger and larger with the mole ratio of AgCl. As we know, a large specific surface area as a photocatalytic material is conducive to adsorption of pollutants [29]. The investigation of Matsui et al. clearly demonstrated that the catalytic activity of ZrO_2_/carbon cluster composite materials increased with the increase of their surface areas [30]. Generally, the performances of semiconductor oxide photocatalysts are dependent on the surface area, mesoporosity, crystallinity, morphology [31], and active facets exposed [32]. The exposed high-index facets (1 1 1) of the AgCl crystals in the Ag@AgCl core–shell nanowires also caused high photocatalytic oxidation efficiency for As(III).

#### Kinetic Study of As(III)

An exponential decay of As(III) concentration with the irradiation time was evident, indicating the photocatalytic oxidation of As(III) was fitted to pseudo-first-order kinetics (Fig. 5b).

1 $$ \hbox{-} \ln \left(\frac{C_t}{C_0}\right)={k}_r K t={k}_{\mathrm{app}} t $$

where *C*_*t*_ is the concentration of As(III) at time *t*, *t* is the reaction time, *C*_0_ is the initial concentration of As(III) solution (mg/L), and the slope *k*_app_ (min^−1^) is the apparent pseudo-first-order reaction rate constant and is calculated from the slope of the plot of −ln(*C*_*t*_/*C*_0_) vs. time.

In this case, *C*_0_ = 2.669 × 10^−5^ mol/L.

The rate constants *k*_app_ (Table 1) of Ag@AgCl core–shell nanowires which were synthesized with different ratios of Ag nanowires:FeCl_3_ from 2.32:0.5 to 2.32:4.0 were increased from 0.009 to 0.023 min^−1^ and then decreased to 0.016 min^−1^. The rate of oxidation (mg L^−1^ min^−1^) was proportional to the rate constant *k*_app_ (min^−1^).

**Table 1:** Pseudo-first-order kinetic parameters of As(III) photocatalytic oxidation

Quantity of FeCl_3_ (mL)	*C*_0_(Exp.)	*C*_0_(cal.)	*R* ^2^	*k*_app_
0.5	1.711	1.586	0.927	−0.009
1.0	1.738	1.555	0.930	−0.010
1.5	1.652	1.489	0.958	−0.010
2.0	1.668	1.565	0.973	−0.015
2.2	1.635	1.494	0.965	−0.023
4.0	1.629	1.406	0.910	−0.016

#### Effects of pH and Ionic Strength

**Fig. 6:**
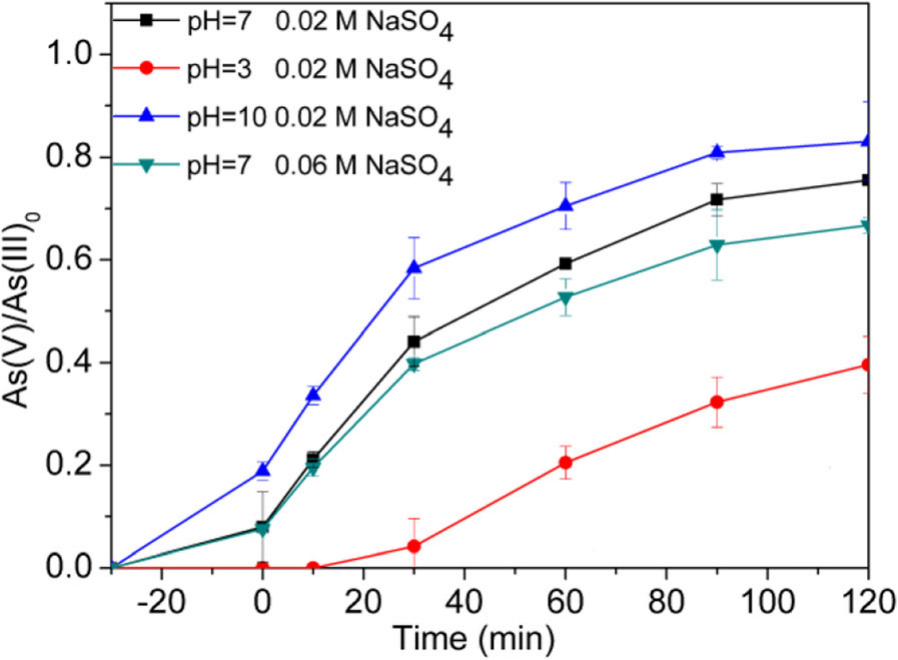
Effects of pH and ionic strength on the photocatalytic oxidation of As(III). Ag@AgCl core–shell nanowires synthesized with the mole ratio of Ag nanowires:FeCl_3_ = 2.32:2.20, and the experimental conditions were [Ag@AgCl] = 0.3 g/L, [As(III)]_0_ = 2.0 mg/L, initial pH = 7.0, and air-equilibrated, *P* = 300 W

To investigate the effect of pH on photocatalytic oxidation of As(III) over Ag@AgCl core–shell nanowires, the experiment was carried out at different values of initial pH (3.0, 7.0, and 10.0), with an initial As(III) concentration of 2.00 mg/L. The As(III) oxidation percentages at initial solution pH 3.0, 7.0, and 10.0 in 2 h were found to be 39.60, 75.55, and 83.13%, respectively (Fig. 6). Thus, it was concluded that the conversion rate of As (III) was significantly influenced by pH and increased with the rise of the initial pH. In the acid reaction solution, the reactive radical of ·O_2_^−^ combined with H^+^ to form HO_2_ and then generated H_2_O_2_ (Eqs. 2 and 3). The oxidation activity of H_2_O_2_ for As(III) was less than ·O_2_^−^, which might be the reason why the oxidation of As(III) decreased as the pH of the solution dropped.

2 $$ \cdotp {{\mathrm{O}}_2}^{\hbox{-} }+{\mathrm{H}}^{+}\to\ \mathrm{H}{\mathrm{O}}_2\cdotp $$

3 $$ 2\mathrm{H}{\mathrm{O}}_2\cdotp \to\ {\mathrm{O}}_2+{\mathrm{H}}_2{\mathrm{O}}_2 $$

4 $$ {\mathrm{H}}_3\mathrm{A}\mathrm{s}{\mathrm{O}}_3 + {\mathrm{H}}_2{\mathrm{O}}_2\to\ {\mathrm{H}}_2\mathrm{A}\mathrm{s}{{\mathrm{O}}_4}^{\hbox{-} } + {\mathrm{H}}_2\mathrm{O} + {\mathrm{H}}^{+ }\ \left(\mathrm{when}\;\mathrm{pH}\ \mathrm{is}\ \mathrm{less}\ \mathrm{than}\ 6.76\right) $$

When NaSO_4_ concentrations in As(III) solution was 0.02 and 0.06 M, the conversion percentages of As(III) were 75.55 and 66.75%, respectively. It is known that Na^+^ is an alkaline metal ion and at its maximum oxidation state; therefore, it will not compete as hole scavenger and does not show distinct effect on photocatalytic reaction [33].

#### Effect of Humic Acids

**Fig. 7:**
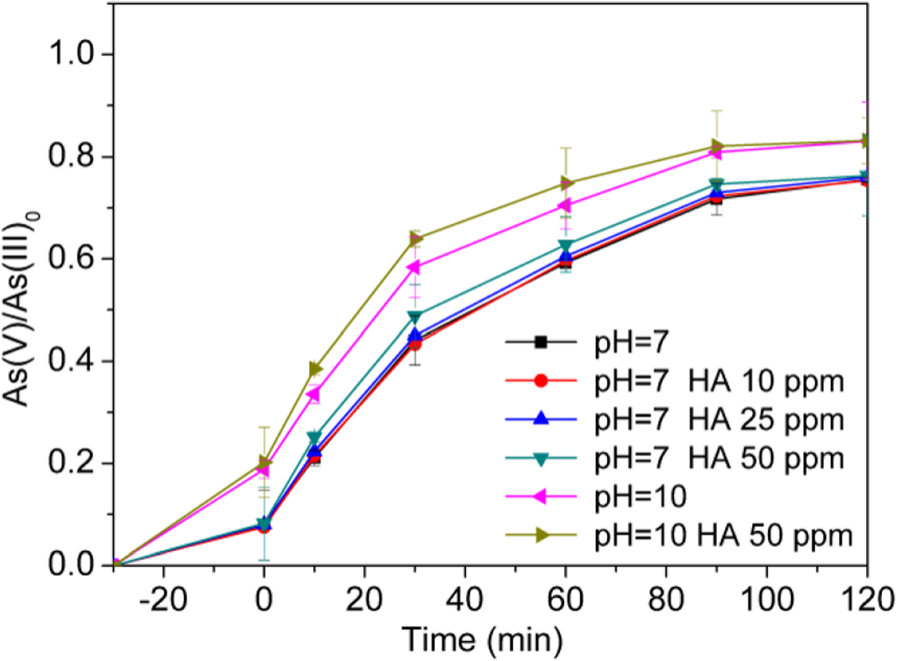
Effect of HA addition on the photocatalytic arsenite oxidation. Ag@AgCl core–shell nanowires synthesized with the mole ratio of Ag nanowires:FeCl_3_ = 2.32:2.20, and the experimental conditions were [Ag@AgCl] = 0.3 g/L, [As(III)]_0_ = 2.0 mg/L, initial pH = 7.0, and air-equilibrated, *P* = 300 W

The concentration of humic substance in natural water varies from 0.03 to 30 mg/L. Arsenic-contaminated groundwater often contains high levels of dissolved organic carbons. The influence of humic acids (HA) on the photocatalytic oxidation of As(III) over Ag@AgCl core–shell nanowires was tested, as shown in Fig. 7. The addition of HA slightly enhanced the oxidation rate of As(III) over Ag@AgCl core–shell nanowires at pH 7.0 and pH 10.0. HA could serve as a hole scavenger which could facilitate the production of superoxides and H_2_O_2_ under visible-light illumination; as the consequence, the As(III) photooxidation rate was enhanced [34]. Therefore, the HA-enhanced effect should be ascribed to the hole scavenging effect.

#### Photocatalytic Mechanism

**Fig. 8:**
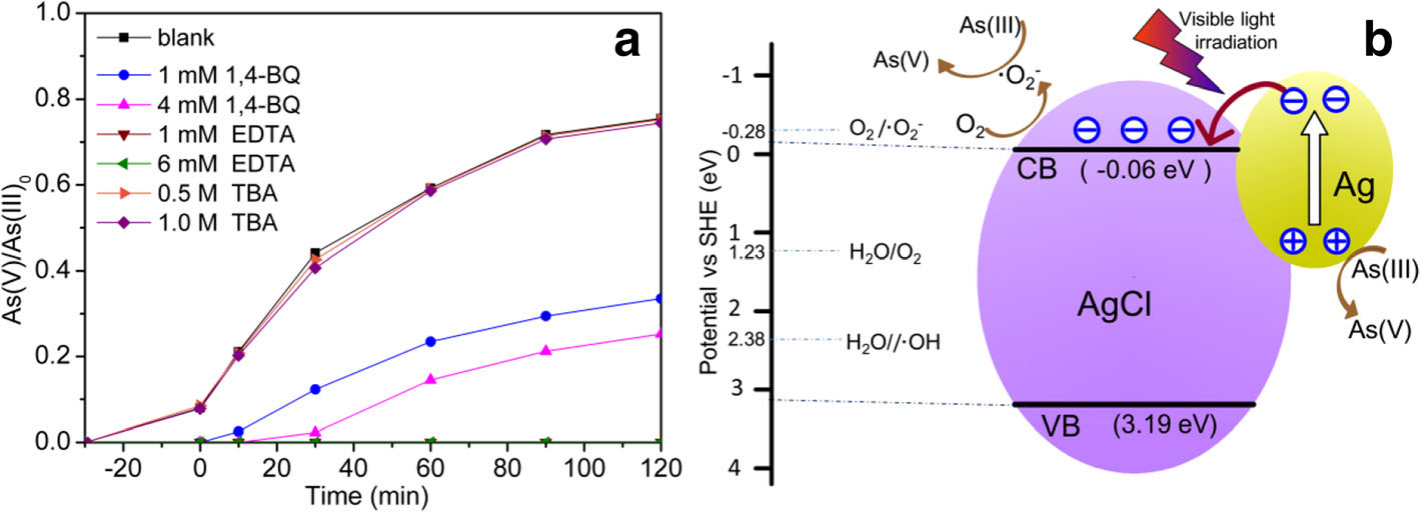
**a** Effects of different scavengers on photocatalytic oxidation of As(III) by Ag@AgCl with 120 min visible-light irradiation. Ag@AgCl core–shell nanowires synthesized with the mole ratio of Ag nanowires:FeCl_3_ = 2.32:2.20, and the experimental conditions were [Ag@AgCl] = 0.3 g/L, [As(III)]_0_ = 2.0 mg/L, and initial pH = 7.0, *P* = 300 W. **b** Schematic diagram of electron–hole pairs separation and the possible reaction mechanism over Ag/AgCl core–shell nanowires under visible-light irradiation

It has been widely accepted that series of active species may be generated to degrade pollutants in photocatalytic process, including superoxide radical (·O_2_^−^), photogenerated holes (h^+^) in the valence bond, and hydroxyl radicals (·OH) [35]. Herein, p-benzoquinone (1,4-BQ) was employed for quenching ·O_2_^−^, tertiary butanol (TBA) for ·OH, and EDTA for h^+^, to study the photocatalytic mechanism of Ag@AgCl (as shown in Fig.8). The photocatalytic oxidation of As(III) over Ag@AgCl core–shell nanowires was not almost affected by the addition of TBA (Fig. 8a), indicating that ·OH was not produced in the photocatalytic reaction system of Ag@AgCl core–shell nanowires. However, the photocatalytic performance was strongly restrained in the presence of BQ or EDTA. The oxidation rate of As(III) was decelerated obviously from 75.55 to 0% in the presence of EDTA(1 mmol/L). It could be elucidated that h^+^ was generated in the photodegradation process and h^+^ was acted as the dominant active species responsible for the photocatalytic oxidation of As(III) under visible-light irradiation. The oxidation rate of As(III) was reduced to 25.25% by 1,4-BQ(4 mmol/L) addition, indicating that ·O_2_^−^ played an important role in the photocatalytic process. Therefore, it could be concluded that h^+^ and ·O_2_^−^ were the main active species for the photocatalytic oxidation of As(III) on Ag@AgCl core–shell nanowires under visible-light irradiation.

In a hybrid composite, the band positions of individual components are crucial to determine the excitation, migration, and recombination of the photogenerated electrons and holes, which is also important for the efficiency of the photocatalytic activity. The valence band (VB) and conduction band (CB) potentials of Ag@AgCl at the point of zero charge are calculated by the following empirical Eqs. (5) and (6):

5 $$ {E}_{vb}= X-{E}_0+0.5{E}_g $$

6 $$ {E}_{cb}={E}_{vb}-{E}_g $$

Where *E*_vb_ is the VB edge potential, *X* is the absolute electronegativity of the semiconductor, *E*_0_ is the energy of free electrons on the hydrogen scale (ca. 4.5 eV), and *E*_g_ is the band gap energy of the semiconductor. Thus, the CB and VB potentials of AgCl are estimated to be −0.06 and 3.19 eV, respectively [36].

Based on the above observations and the literature, the possible transfer routes of the photoexcited electrons and holes and a feasible photocatalytic oxidation mechanism for As(III) in the presence of Ag@AgCl core–shell nanowires under visible light are proposed and schematically illustrated in Fig. 8b. Because AgCl has a direct band gap of 5.15 eV (241 nm) and an indirect bandgap of 3.25 eV (382 nm) [37], it cannot be excited under the illumination of visible light (*λ*ex > 420 nm). However, metallic Ag nanoparticles (Ag NPs) can absorb a photon under visible-light irradiation leading to electron–hole (e^−^–h^+^) separation because of its strong intrinsic plasmon resonance (SPR) effect (Eq. (7)). The photoinduced electrons transiently occupy the CB of Ag and then sequentially transfer to the CB of AgCl, while the plasmon-induced holes (h^+^) remained on the surface of Ag NPs. This electrons transfer process can effectively promote the charge separation and inhibit the recombination of the electron–hole pair. Since the CB energy level of AgCl (−0.06 V vs NHE) is more negative than the potential of O_2_/·O_2_^−^ (−0.046 V vs NHE), the electrons in the CB of AgCl will be trapped by the surface adsorbed molecular O_2_ to generate ·O_2_^−^ (Eq. (8); [38]). ·O_2_^−^ is a kind of active species with strong oxidation power that can convert As(III) to As(V) (Eqs. (11) and (12)). In the meantime, a few electrons combine with Ag^+^ ions to form Ag^0^ atoms with the lixiviation of chloridion (Cl^−^), which is another competing trapping route for the photoinduced electrons (Eq. (9); [17]). Plasmon-induced h^+^ on the surface of Ag NPs can directly oxidize As(III) to As(V) (Eq. (13)). It is possible that Cl^−^ on the surface of AgCl can be oxidized to Cl^0^ atoms by some of the plasmon-induced h^+^ on the surface of Ag NPs (Eq. (10)). Cl^0^ atom is a kind of reactive radical species that can efficiently oxidize As(III) and be reduced to Cl^−^ again (Eq. (14); [28, 39]).

7 $$ \mathrm{AgNPs}\left(\mathrm{SPR}\right) + \mathrm{h}\mathrm{v}\to {\mathrm{e}}^{\hbox{--}}\left(\mathrm{electron}\right) + {\mathrm{h}}^{+}\left(\mathrm{hole}\right) $$

8 $$ {\mathrm{O}}_2 + {\mathrm{e}}^{\hbox{-}}\to \cdotp {{\mathrm{O}}_2}^{\hbox{-} } $$

9 $$ \mathrm{A}\mathrm{gCl} + {\mathrm{e}}^{\hbox{-}}\to \mathrm{A}{\mathrm{g}}^0 + \mathrm{C}{\mathrm{l}}^{\hbox{-} } $$

10 $$ {\mathrm{h}}^{+}+\mathrm{C}{\mathrm{l}}^{\hbox{--}}\to \mathrm{C}{\mathrm{l}}^0 $$

11 $$ {\mathrm{H}}_3\mathrm{A}\mathrm{s}{\mathrm{O}}_3+{\mathrm{H}}_2{\mathrm{O}}_2\to\ {\mathrm{H}}_2\mathrm{A}\mathrm{s}{{\mathrm{O}}_4}^{\hbox{-} } + {\mathrm{H}}_2\mathrm{O} + {\mathrm{H}}^{+}\left(\mathrm{pK}{\mathrm{a}}_2 = 6.76,\mathrm{when}\kern0.24em \mathrm{pH}\ \mathrm{is}\ \mathrm{less}\ \mathrm{than}\ 6.76\right) $$

12 $$ 3{\mathrm{H}}_3\mathrm{A}\mathrm{s}{\mathrm{O}}_3 + 2\cdotp {{\mathrm{O}}_2}^{\hbox{-} } + 4\mathrm{O}{\mathrm{H}}^{\hbox{-}}\to\ 3\mathrm{HAs}{{\mathrm{O}}_4}^{2\hbox{-} } + 5{\mathrm{H}}_2\mathrm{O}\ \left(\mathrm{pK}{\mathrm{a}}_2 = 6.76,\mathrm{when}\kern0.24em \mathrm{pH}\ \mathrm{is}\ \mathrm{greater}\ \mathrm{than}\ 6.76\right) $$

13 $$ 2{\mathrm{h}}^{+} + \mathrm{A}\mathrm{s}\left(\mathrm{III}\right)\to\ \mathrm{A}\mathrm{s}\left(\mathrm{V}\right) $$

14 $$ 2\mathrm{C}{\mathrm{l}}^0 + \mathrm{A}\mathrm{s}\left(\mathrm{III}\right)\to\ \mathrm{C}{\mathrm{l}}^{\hbox{-} } + \mathrm{A}\mathrm{s}\left(\mathrm{V}\right) $$

#### Recycling

**Fig. 9:**
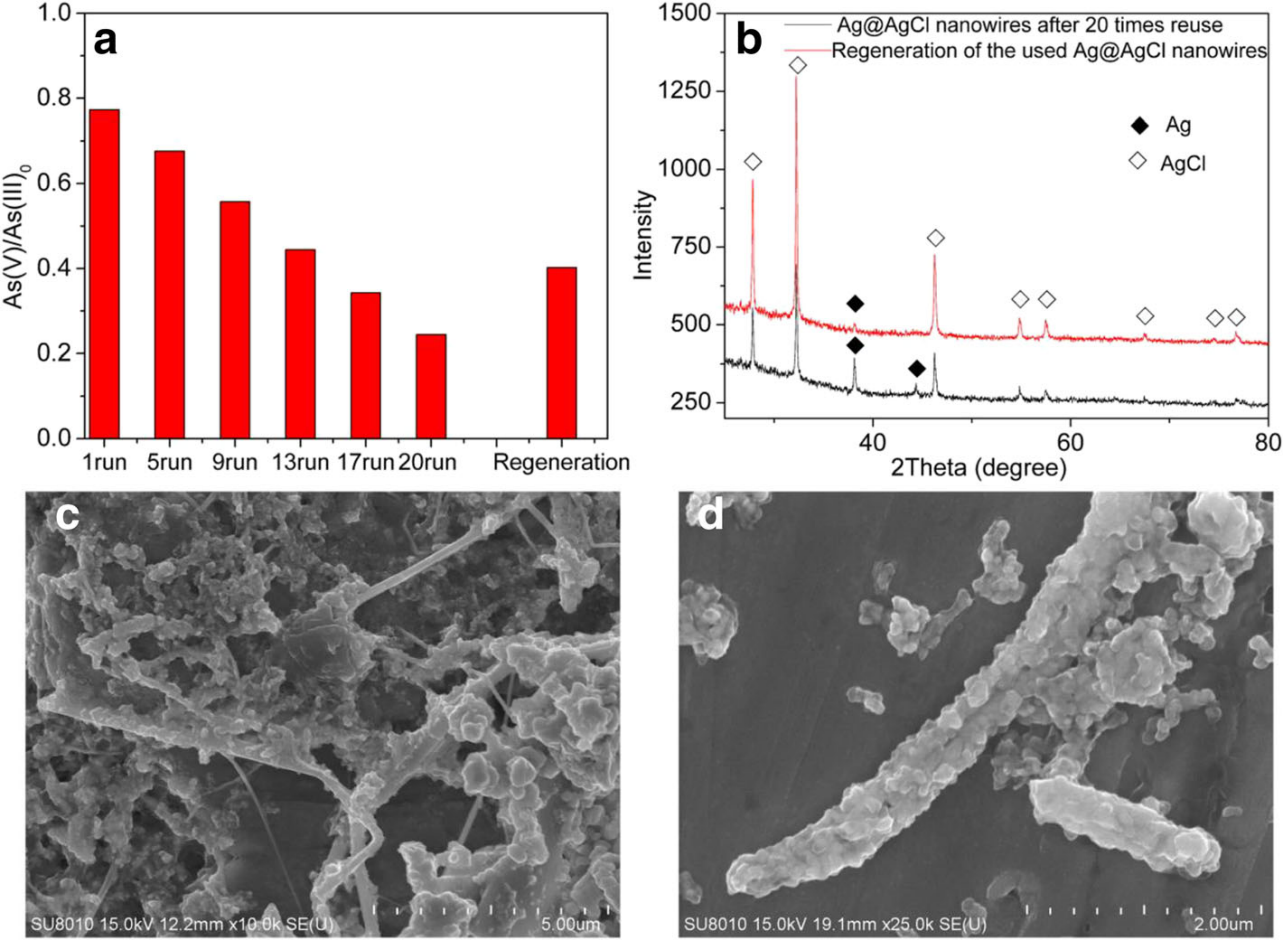
**a** Photostability test of the Ag@AgCl for 20 cycles and regenerated Ag@AgCl. **b** XRD patterns of Ag@AgCl after the 20th cycle and regenerated Ag@AgCl. **c** SEM images of Ag@AgCl after the 20th cycle. **d** SEM images of regenerated Ag@AgCl

In addition to efficiency, stability and recyclability of practical photocatalysts are also important for applications. As we know, lifetime of photocatalysts is an important factor for practical application as well as its photocatalytic activity. In order to test the stability and reusability, Ag@ AgCl core–shell nanowires synthesized with Ag nanowires:FeCl_3_ = 2.32:2.20 were selected as the model photocatalysts to test the repeatability of As(III) oxidation and were reused for photocatalytic oxidation of As(III) 20 times with same conditions. Results are shown in Fig. 9a. The oxidation percentage of As(III) decreased from 77.3% in the 1st run to 22.4% in the 20th cycle. The decrease of the oxidation efficiency was due to some catalyst washout during the recycling steps.

To better understand the decrease reason of photocatalytic oxidation efficiency during recirculation process, the samples of Ag@AgCl core–shell nanowires after 20 cycles of photocatalytic oxidation for As(III) were tested by XRD and SEM. It was found that the diffraction peak of Ag^0^ at 38.2° with enhanced intensity could be observed in the XRD pattern of the sample after the twentieth cycle (Fig. 9b). This revealed the continuous transformation from AgCl into Ag^0^ with the leaching of chloridion (Cl^−^) during the recycling process. Such kind of transformation led to a deviation from the optimum ratio of AgCl to Ag with the best photocatalysis performance, which should be responsible for the continuous and slight decrease of photocatalysis activity in the recycle runs. What is more, the formation of Ag nanoparticles resulted in the decrease of the interface area between Ag and AgCl, which was another reason for the decrease of photocatalytic activity. This phenomenon was also found in other Ag@AgCl nanocomposites [39]. The SEM image (Fig. 9c) showed that the core–shell nanowires cracked during the photocatalytic activity.

The 20th recycled Ag@AgCl nanowires were collected and reacted with FeCl_3_ solution again; the SEM image showed that Cl-afresh core–shell nanowires structure was rebuilt, but cracked more seriously which might be caused in the process of ultrasonic dispersion, and became thicker because of the agglomeration (Fig. 9d). The The XRD patterns showed that the mole ratio of Ag:AgCl in regenerated Ag@AgCl core–shell nanowires returned the optimum again (Fig. 9b). The As(III) oxidation rate of regenerated Ag@AgCl core–shell nanowires was increased to 40.2% (Fig. 9a), which proved Cl-afresh of recycled Ag@AgCl core–shell nanowires could efficiently prolong the lifetime of prepared photocatalysts.

## Conclusions

In order to exploit more visible-light-responding photocatalysts for As(III) oxidation, Ag@AgCl core–shell nanowires synthesized via a controllable oxidation reaction with different mole ratios of Ag nanowires:FeCl_3_ in solution were selected to conduct the photocatalytic oxidation experiment for As(III). Ag@AgCl core–shell nanowires showed excellent photocatalytic activity toward As(III) oxidation and the mole ratio of Ag nanowires:FeCl_3_ had obvious influence on its photocatalytic ability. Photocatalytic oxidation rate of As(III) was favored at high pH and could be promoted by humus acid. High concentration of Na_2_SO_4_ in solution will slightly inhibit the photocatalytic oxidation reaction. With quenching agent, the holes and ·O_2_^−^ were proved to be the main active materials in the photocatalytic oxidation process for As (III). The photocatalytic ability of Ag@AgCl core–shell nanowires gradually decreased with recycle times, and Ag@AgCl core–shell nanowires after recycling could be efficiently regenerated by Cl-afresh. The prepared Ag@AgCl core–shell nanowires were proved to be an efficient and relatively stable visible-light-induced photocatalyst for As(III) oxidation in water with high humic substance.
